# Inter-rater reliability of psychiatric diagnosis: a systematic review and metanalysis

**DOI:** 10.1192/j.eurpsy.2025.457

**Published:** 2025-08-26

**Authors:** C. L. Di Forti, D. Liccione, C. Scarpazza

**Affiliations:** 1 Department of Brain and Behavioral Sciences, University of Study of Pavia, Pavia; 2 Department of Mental Health and Addiction, ASST Santi Paolo e Carlo, Milano; 3 Department of General Psychology, University of Study of Padova, Padova, Italy

## Abstract

**Introduction:**

Psychiatric diagnosis plays a key rol in the mental health care. One of the critical factors that influence the diagnostic process is inter-rater reliability, the degree to which different raters agree on the diagnosis when assessing the same patient. Despite the availability of standardized diagnostic manuals, variability in psychiatric diagnoses persists. The assessment of inter-rater reliability involves calculating statistical measures which quantify the level of agreement between raters beyond what would be expected by chance. Improving inter-rater reliability in psychiatric diagnoses is necessary for optimizing both patient care and research quality in mental health.

**Objectives:**

Assess inter-rater reliability across main psychiatric disorders and identify the sources of variability.

**Methods:**

This study was performed according to the PRISMA guidelines and a total of ninety-three studies were included. Regarding inclusion criteria, (1) the articles had to focus on inter-rater reliability, (2) study participants had to have an average age greater than 18 years, and (3) the reported diagnoses had to refer to a diagnostic manual. Quality scores were assessed for all included studies (Armijo-Olivo S *et al.* J Eval Clin Pract. 2012; 18 12-8). Seven different meta-analysis were conducted, one for each psychiatric diagnosis detected. The heterogeneity between studies was quantified using Cochran’s *Q* and *I* ^2^. Funnel plots was analyzed to assess the possible influence of publication and location biases (Higgins&Green. BMJ. 2011;343). To account for publication bias, the Eggers’ test and the Fail-Safe Number31 was applied.

**Results:**

**Psychotic disorder:** (k=0.70; 95% CI: 0.66-0.75) (I²=97%).

**Anxiety Disorders:** (k=0.65; 95% CI: 0.60-0.70) (I²=91%).

**Obsessive-Compulsive Disorder (OCD):** (k=0.73; 95% CI: 0.64-0.82) (I²=76%)

**PTSD:** (k=0.60; 95% CI: 0.52-0.66) (I² =84%).

**DCA:** (k=0.73; 95% CI: 0.67-0.79) (I² =76%).

**Personality disorder (PD):**

two different meta-analysis were conducted because many studies used Intraclass Correlation Coefficient (ICC) as a value to express inter-rater agreement. [k=0.65 (95% CI: 0.59, 0.7)] (I^2^= 96%) (ICC=0.85; 95% CI: 0.82-0.87) (I² = 66%).

**Image 1:**

**Figure:**
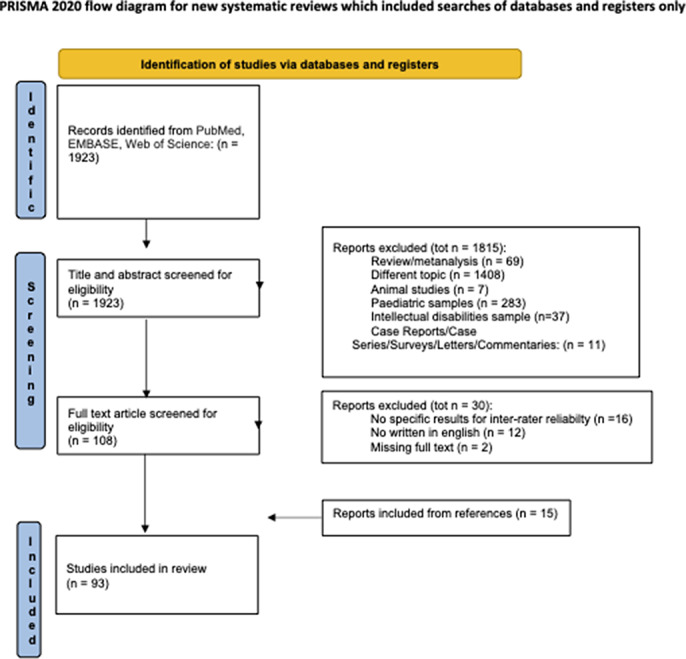

**Conclusions:**

The lower Kappa for s**chizoaffective disorder** (Kappa < 0.7) compared to other psychiatric disorders underscores the diagnostic challenges posed by this category, given its overlapping symptoms with both mood and psychotic disorders. As regards personality disorder, **antisocial and borderline PD** showed highest agreement potentially due to its well-defined diagnostic criteria. The lowest agreement (k=0.60) of **PTSD** emphasizes the variability of his clinical presentation. In conclusion, studies show variability across disorders, highlighting the need for further research to improve diagnostic accurac (Regier *et al.* Am J Psychiatry. 2009;166 645-50) thereby enhancing clinical and research outcomes.

**Disclosure of Interest:**

None Declared

